# A Nosocomial Outbreak of *Burkholderia cepacia complex* Linked to Contaminated Intravenous Medications in a Tertiary Care Hospital

**DOI:** 10.3390/antibiotics14080774

**Published:** 2025-07-31

**Authors:** Hanife Nur Karakoc Parlayan, Firdevs Aksoy, Masite Nur Ozdemir, Esra Ozkaya, Gurdal Yilmaz

**Affiliations:** 1 Department of Infectious Disease and Clinical Microbiology, Faculty of Medicine, Karadeniz Technical University, 61080 Trabzon, Türkiye; faslanaksoy@ktu.edu.tr (F.A.); masitenurozdemir@ktu.edu.tr (M.N.O.); gurdalyilmaz@ktu.edu.tr (G.Y.); 2 Department of Medical Microbiology, Faculty of Medicine, Karadeniz Technical University, 61080 Trabzon, Türkiye; esraozkaya@ktu.edu.tr

**Keywords:** *Burkholderia cepacia complex*, outbreak, interventional procedures, contaminated medications

## Abstract

Objectives: *Burkholderia cepacia complex* (Bcc), a Gram-negative organism, is a well-recognized cause of hospital outbreaks, often linked to a contaminated shared source, such as multidose medications. In this study, we report an outbreak of Bcc infections in a tertiary care hospital, associated with the intrinsic contamination of a prepared solution used in interventional radiology (IR) procedures. Additionally, we provide a detailed explanation of the interventions implemented to control and interrupt the outbreak. Methods: Records from the infection control committee from 1 January 2023 to 31 October 2024 were screened to identify cases with Bcc growth in cultured blood, urine, or respiratory samples. Clinical and laboratory data were collected in March 2025. Bacterial identification was performed using conventional methods and MALDI-TOF (Bruker Daltonics, Bremen, Germany). Controls were matched to cases by ward, date of initial growth, and duration of hospitalization. Demographic and clinical data of these patients were systematically collected and analyzed. Microbiological cultures were obtained from environmental objects of concern and certain medications. Results: A total of 82 *Burkholderia* species were identified. We enrolled 77 cases and 77 matched controls. The source of contamination was identified in ready-to-use intravenous medications (remifentanil and magnesium preparations) in the IR department. These preparations were compounded in advance by the team and were used repeatedly. Although the outbreak originated from contaminated IV medications used in IR, secondary transmission likely affected 28 non-IR patients via fomites, shared environments, and possible lapses in isolation precautions. The mortality rate among the cases was 16.9%. Infection with Bcc was associated with prolonged intensive care unit stays (*p* = 0.018) and an extended overall hospitalization duration (*p* < 0.001); however, it was not associated with increased mortality. The enforcement of contact precautions and comprehensive environmental decontamination successfully reduced the incidence of the Bcc outbreak. No pathogens were detected in cultures obtained after the disinfection. Conclusions: The hospital transmission of Bcc is likely driven by cross-contamination, invasive medical procedures, and the administration of contaminated medications. Implementing stringent infection control measures such as staff retraining, updated policies on medication use, enhanced environmental decontamination, and strict adherence to isolation precautions has proven effective in curbing the spread of virulent and transmissible Bcc.

## 1. Introduction

*Burkholderia cepacia complex* (Bcc), is an aerobic, non-fermentative, Gram-negative bacillus that is oxidase-positive and catalase-positive. It is widely distributed in natural environments, including soil and water, and can also exist in plants, either as phytopathogens or as biocontrol agents [1,2].

Bcc is an opportunistic pathogen that rarely causes infections in immunocompetent individuals but poses a significant risk to patients with cystic fibrosis (CF), chronic granulomatous disease, and immunosuppression. It can cause a wide range of infections in non-CF individuals, particularly impacting vulnerable groups. These include those with chronic or critical illnesses and immunocompromised individuals, where it can manifest as pneumonia, meningitis, urinary tract infections, and bloodstream infections (BSIs) [3,4].

Additionally, it is associated with outbreaks in patients dependent on medical devices or those receiving prolonged hospital care. Its ability to form biofilms on medical devices further exacerbates its persistence in hospital environments, making infection control challenging [5]. It also facilitates colonization of the respiratory tract and prosthetic devices, such as intravascular catheters. The outbreaks often arise from shared use of contaminated medical products such as disinfectants, saline solutions, or respiratory therapy equipment, reflecting the organism’s adaptability and intrinsic resistance to antimicrobial agents and disinfectants [6,7].

In healthcare settings, Bcc presents significant challenges due to its intrinsic resistance to multiple antibiotics and disinfectants. This resistance is often mediated by efflux pumps, decreased outer membrane permeability, and the presence of β-lactamases [8]. It has been linked to significant morbidity and mortality, particularly among immunocompromised patients or those with underlying conditions. It is a critical concern in healthcare settings, known for causing severe infections and outbreaks in these vulnerable populations [9,10].

The bacterium’s multidrug-resistant nature limits treatment options, often requiring combination antibiotic therapies [8,11]. Furthermore, its ability to survive in environments with minimal nutrients enables its prolonged persistence in healthcare settings, leading to repeated outbreaks [12].

We report a healthcare-associated outbreak involving 77 patients at a 700-bed tertiary care university hospital in Türkiye. The investigation identified the source of the outbreak as a contaminated liquid drug prepared for invasive procedures in the IR department. Following this discovery, the contaminated source was removed, and the area underwent thorough disinfection to prevent further cases.

## 2. Results

Between 1 January 2023 and 31 October 2024, a total of 82 *Burkholderia* species were initially evaluated for eligibility in this study. Of this group, 77 were *Burkholderia cepacia*, 4 were *Burkholderia gladioli*, and 1 was *Burkholderia cenocepacia*. *Burkholderia gladioli* and *Burkholderia cenocepacia* were excluded from the analysis due to the missing data (Table 1).

Of the 77 patients, 42 (54.5%) were male and 35 (45.5%) were female. The overall mean age was 40.2 ± 20.7 years (range: 0–90). The most common types of growth were observed blood cultures (*n*: 59; 76.6%), followed by respiratory samples (*n*: 8; 10.4%), urinary samples (*n*: 7; 9.1%), and abscess drainage samples (*n*: 3; 3.9%). Bcc was identified as the causative pathogen in 57 cases (74%), prompting the commencement of targeted antimicrobial therapy. Of these, 48 cases (84.2%) were managed with a diagnosis of bacteremia.

The mean duration of hospitalization was 26.3 ± 20.8 days (range: 0–114), with culture samples obtained on average 6.5 ± 7.8 days (range: 0–39) after admission. The mortality rate among the cases was 13 (16.9%). The presence of Bcc in sample cultures did not increase mortality rates (*p* = 0.652) compared to the control group. However, it was associated with prolonged intensive care unit (ICU) stays (median duration, 7 vs. 2 days; *p* = 0.018) and extended total hospitalization duration (median duration, 22 vs. 6 days; *p* < 0.001).

**Figure 1:**
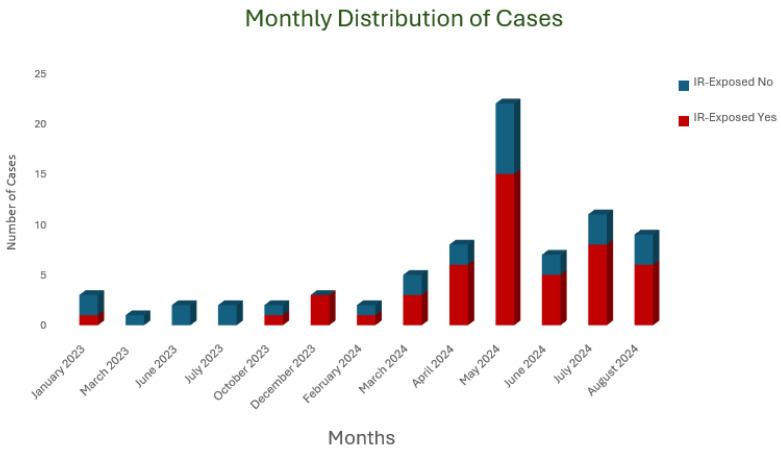
Monthly distribution of *Burkholderia cepacia complex* cases during outbreak.

The monthly distribution of cases is illustrated in Figure 1, highlighting an outbreak that occurred between January 2023 and August 2024. The outbreak drew our attention in May 2024, with the highest number of cases reported, prompting the initiation of our investigation. In response, hospital-wide awareness efforts, environmental screening, and staff retraining were undertaken.

The analysis of transmission risk factors is presented in Table 2. A substantial proportion of patients—49 (63.6%)—had undergone interventional radiology procedures, 47 (61%) had a history of ICU admission, and 41 (53.2%) had a history of surgery. The study revealed that, compared to the control group, central catheter placement and IR procedures were identified as the highest risk factors. In response, inspections were conducted in intensive care units, operating rooms, and IR suites. Environmental samples were collected from potential outbreak sources, including disinfectants, mechanical ventilation devices, and other medical equipment.

The source of contamination was identified as ready-to-use intravenous medications in IR. The presence of Bcc was detected in remifentanil and other medical preparations containing magnesium, which were prepared in advance and used by the IR team. These medications were prepared by the unit’s team before the first procedure of the day and were used repeatedly throughout the day until the vials were depleted. These preparations were not distributed or used outside the interventional radiology department.

Following the identification of contamination sources in August 2024, immediate mitigation steps were taken. The infection control committee conducted target inspections and provided corrective training to the technical and nursing staff. The IR unit was temporarily closed, decontamination procedures were implemented, and IR personnel underwent additional training and were closely monitored. In addition, a new protocol was implemented, and all intravenous medications are now prepared as single-use doses, with each vial dedicated to a single patient only.

After the implementation of contact precautions and comprehensive environmental decontamination, the Bcc outbreak was successfully controlled, with previously identified samples and environmental cultures testing negative for Bcc. Since then, no new documented cases have been detected.

## 3. Discussion

This study describes a prolonged outbreak of Bcc in a tertiary university hospital involving a significant number of cases (77 patients) and attributed to the reuse of previously compounded intravenous medications. It underscores the critical role of contaminated intravenous preparations in nosocomial outbreaks caused by Bcc, highlighting its impact on patient outcomes and the importance of stringent infection control measures.

This study highlights the critical importance of sustaining heightened awareness for potential outbreaks, particularly in the presence of uncommon pathogens such as Bcc, to enable timely detection and effective intervention. It further highlights the essential role of infection control surveillance in uncovering the source of an outbreak.

Bcc is a recognized pathogen responsible for nosocomial outbreaks, primarily affecting the bloodstream and respiratory tract. These outbreaks are often linked to a common contaminated source, including disinfectants, aqueous solutions, liquid reservoirs, and pharmaceuticals [6,7,13,14]. Similarly, in our study, Bcc was identified in 76.6% of blood samples, followed by respiratory samples (10.4%), consistent with findings reported in the literature.

Previous investigations have identified invasive procedures as significant risk factors for Bcc infections, including central venous catheters (CVCs), hemodialysis, mechanical ventilation, and recent surgical interventions [15,16,17]. Similarly, a study conducted in a neonatal intensive care unit highlighted CVCs as a major risk factor for nosocomial Bcc infections [18]. Lee et al. also mention in their study that catheter-related infection as a source of bacteremia was associated with increased mortality in patients with Bcc bacteremia [15]. Our study demonstrated that IR procedures were linked to an 11.7-fold increase in the presence of Bcc compared to the control group. The presence of Bcc was detected in intravenous medications, such as remifentanil, used in the IR department. Although IR was identified as the primary source of the outbreak using contaminated intravenous medications, 28 patients without documented interventional radiology exposure also developed Bcc infections. We interpret that these patients had shared clinical environments with exposed interventional radiology cases, particularly in intensive care units, suggesting the possibility of indirect transmission through shared equipment, healthcare personnel, or residual environmental contamination prior to outbreak containment.

Several outbreaks of Bcc have been reported worldwide, linked to pharmaceuticals with intrinsic resistance, such as nasal sprays [19], liquid docusate laxatives [20], or medications prepared and reused throughout the day [10]. Other sources include medical devices [21], personal care products [7,12], and decontamination solutions used in intensive care units [22]. Ormsby et al. [23] emphasized that adherence to standardized protocols for medication preparation, including the use of single-dose vials, preparation in sterile environments, and strict aseptic technique, are critical in preventing such incidents. In our case, although the medications were not officially designated as multidose vials, they were compounded at the beginning of the day and used repeatedly across multiple patients, which effectively constituted multidose use. Contamination is presumed to have occurred either during the preparation process or due to prolonged storage under non-sterile conditions. This highlights a critical infection control lapse, distinct from device-related transmission, and underscores the need for strict aseptic technique and adherence to single-use medication protocols in high-risk settings such as IR.

International guidelines recommend structured staff training, rigorous environmental decontamination, and strict implementation of standard and transmission-based precautions as core components of effective infection prevention and control programs [24,25]. In line with these guidelines, the outbreak was successfully controlled following the implementation of contact precautions, comprehensive environmental decontamination using hospital-grade bleach and chlorine-based disinfectants, and staff education programs. Identifying the outbreak source and conducting a thorough epidemiological investigation led to significant improvements in infection prevention measures throughout our facility. These measures included increasing the frequency of environmental care rounds and transitioning to the use of single-dose sterile IV medications for procedures.

In alignment with the existing literature, this outbreak further emphasizes the role of invasive procedures, including central venous catheterization, as significant risk factors for Bcc transmission. However, the rapid enforcement of contact precautions and environmental decontamination demonstrated the effectiveness of stringent infection control measures. Notably, no pathogens were detected in follow-up cultures after these interventions, reinforcing the importance of proactive outbreak management.

In the literature, mortality rates for Bcc infections have been reported to range between 16% and 60% [4,9,22]. In our study, the mortality rate among cases with Bcc presence was found to be 17%. Similarly, a study investigating cases of Bcc bacteremia reported that early removal of CVCs reduced mortality to 16%, whereas delayed or no removal resulted in a higher mortality rate of 29%, consistent with prior reports [15]. In our cohort, 52% of cases had CVCs, and timely catheter removal was performed in bacteremic patients to ensure source control. This proactive intervention likely contributed to the relatively lower mortality rate observed in our study.

This study found no significant association between the presence of Bcc and mortality compared to the control group. However, the matched case–control design facilitated a comparison, demonstrating that Bcc presence extended ICU stays and overall hospitalization duration. Clinical outcome studies indicate that patients infected with Bcc are managed with various therapeutic regimens, leading to prolonged hospital stays [15,17,26,27]. These findings are consistent with prior studies that associate Bcc infections with increased healthcare resource utilization and elevated patient morbidity. The delayed detection of this outbreak likely contributed to the high number of affected patients and the associated morbidity. The prolonged recognition period can be attributed to the sporadic distribution of cases across various hospital units and the non-specific clinical manifestations of Bcc infections. In response, microbiological surveillance was enhanced through the integration of automated alert systems for rare pathogens and abnormal clustering patterns. A structured communication pathway was established between the microbiology laboratory, infection control team, and pharmacy to ensure timely notification and coordinated investigation of potential outbreaks. The personnel in high-risk areas such as IR and the ICU were re-trained in aseptic techniques, safe medication handling, and infection prevention protocols. To prevent recurrence, all intravenous medications are now prepared as single-use, patient-specific doses, and the reuse of pre-compounded medications has been strictly discontinued.

## 4. Materials and Methods

### 4.1. Study Design and Setting

This cross-sectional study was conducted between 1 January 2023 and 30 October 2024 in a tertiary university hospital in Türkiye. The hospital provides services across multiple units, including an ICU, a burn center, inpatient wards, outpatient clinics, and the emergency department. Records from the infection control committee were retrospectively screened to identify cases included in the study, specifically those with Bcc isolates detected in cultures from blood, urine, sputum, deep tracheal aspirate (DTA), or bronchoalveolar lavage samples (BAL).

Laboratory and clinical data of patients were extracted from the electronic medical records system. The collected information included demographic details, clinical presentations, comorbidities, potential risk factors, clinical progression (such as admission to critical care), infection sites, microbiological findings, and in-hospital mortality. In this study, which focused on patients with Bcc isolates, a control group was formed to identify the outbreak source.

### 4.2. Study Population

A case was defined as any patient who presented to Karadeniz Technical University Farabi Hospital between 1 January 2023 and 30 October 2024 and had *Burkholderia* spp. isolates identified in sputum, blood, urine, DTA, or BAL cultures.

Controls were defined as patients who were hospitalized in the same ICU or ward as their matched cases during the outbreak period (January 2023–October 2024) who did not have any Bcc isolated from clinical specimens. Matching was performed in a 1:1 ratio based on hospitalization unit, admission date proximity (within the same month), age group, sex, and similar clinical indication for hospitalization (e.g., respiratory failure, sepsis, or post-operative care). Controls were confirmed to have had at least one relevant culture (e.g., blood, sputum, urine, DTA, or BAL) submitted during hospitalization, all of which were negative for Bcc.

### 4.3. Data Collection

The data were accessed for research purposes from 4 March 2025 to 14 March 2025. For each case, a single Bcc isolate was included in the analysis, selected based on the infected site and/or the first positive culture identified during the hospitalization. Data collected included patient demographics (age, sex), comorbidities, length of hospital stay, time from hospital admission to Bcc isolation, history of ICU admission, duration of ICU stay, use of invasive devices (such as thoracic tubes, invasive or noninvasive mechanical ventilation, central venous catheters, and nasogastric tubes), history of invasive procedures (including tracheostomy, bronchoscopy, and thoracentesis), history of surgical procedures within the last three months, and the presence of polymicrobial growth in the same culture.

An assessment of potential environmental reservoirs for Bcc was systematically conducted during the outbreak period. Environmental sampling was performed in the rooms of affected patients, with priority given to high-touch surfaces, reusable medical equipment (e.g., mechanical ventilators), and commonly used disinfectants to identify the origin of the contamination. The selection of sampling sites was based on epidemiological links between patients and shared healthcare equipment. Sampling and culture protocols followed hospital infection control guidelines, using sterile swabs and transport media suitable for gram-negative organisms. Furthermore, after analyzing medications common to the cases, pharmaceutical product cultures were initiated with these medications. Although no formal investigation was conducted at the manufacturer level, the findings were reported to the hospital pharmacy and relevant regulatory authorities for further action.

### 4.4. Bacterial Identification

Blood cultures were processed using the BD BACTEC™ FX automated blood culture system (Becton Dickinson Microbiology System, Franklin Lakes, NJ, USA). Following the manufacturer’s instructions, BACTEC blood culture bottles were inoculated with patient blood samples and incubated until a positive signal was detected. Upon positivity, the bottles were removed from the system, and smears were prepared for Gram staining [28]. Subsequently, samples were streaked onto 5% sheep blood agar (Becton Dickinson, Franklin Lakes, NJ, USA), eosin methylene blue agar (Oxoid, Basingstoke, UK), and chocolate agar (Becton Dickinson, Franklin Lakes, NJ, USA) [29].

Respiratory tract specimens, including sputum, DTA, and BAL samples, were also inoculated onto the aforementioned media following established protocols [30,31]. Urine specimens were similarly cultured on 5% sheep blood agar and eosin methylene blue agar. All inoculated plates were incubated at 35–37 °C for 24 to 48 h. Bacterial isolates were identified using conventional microbiological methods and MALDI-TOF mass spectrometry (Bruker Daltonics, Bremen, Germany) according to the manufacturer’s instructions.

### 4.5. Antimicrobial Susceptibility Testing

As the European Committee on Antimicrobial Susceptibility Testing (EUCAST) has not established species-specific breakpoints for Burkholderia cepacia complex (Bcc) organisms, antimicrobial susceptibility testing was conducted and interpreted based on the clinical breakpoints defined for Burkholderia pseudomallei, a taxonomically related species within the same genus (EUCAST, Version 15.0) [32]. Accordingly, susceptibility to selected antimicrobial agents—including ceftazidime, meropenem, tetracycline, and trimethoprim-sulfamethoxazole—was assessed using the standard disk diffusion method.

The study adhered to the ethical standards described in the Declaration of Helsinki and was approved by the Clinical Research Ethics Committee of Karadeniz Technical University (approval no: 2024/275). Informed consent was waived due to the retrospective nature of the study, and anonymous clinical data were used in the analysis.

### 4.6. Data and Statistical Analyses

Data analyses were performed using IBM Statistical Package for the Social Sciences (SPSS), version 23 (IBM Corp., Armonk, NY, USA). Descriptive statistics of cases were expressed using means with standard deviations (SDs). Categorical variables were presented as percentages. Comparisons between groups were assessed with the Chi-square or Fisher’s exact test for categorical variables and a *t*-test for continuous measures.

The strength of associations was assessed using odds ratios (ORs) and the corresponding 95% confidence intervals (CIs) to estimate the relationship between the case and control groups regarding factors contributing to Bcc growth. These factors included the use of various invasive devices (e.g., thoracic tubes, invasive or noninvasive mechanical ventilation, central venous catheters, nasogastric tubes) and histories of invasive procedures (e.g., tracheostomy, bronchoscopy, thoracentesis). A *p*-value of <0.05 was considered statistically significant.

## 5. Limitations of the Study

This study has several strengths, including the identification of a clear source of contamination and the use of matched controls. However, the absence of molecular typing to confirm clonal spread is a limitation. Nevertheless, the approach used in this investigation may serve as a practical guide for identifying outbreak sources in resource-limited settings. Future studies should explore genetic mechanisms of Bcc resistance and assess the effectiveness of alternative decontamination protocols.

## 6. Conclusions

The outbreak was successfully contained through retraining of healthcare personnel, revision of medication preparation and administration policies, enhanced environmental cleaning, and reinforced adherence to isolation precautions.

The contamination of medical devices, antiseptics, or medications with Bcc during preparation can result in severe and potentially life-threatening infections. Raising awareness among healthcare workers about the potential contamination of pharmaceutical products, particularly ready-to-use intravenous medications that are pre-compounded and used repeatedly, could facilitate the early detection of such outbreaks. In these situations, the Infection Control Department plays a pivotal role in prevention and management efforts. The use of multidose vials requires strict adherence to infection control policies and standards to effectively minimize the risk of cross-contamination of medications.

The findings of this study highlight the critical need for vigilant infection control practices, stringent pharmaceutical handling protocols, and ongoing surveillance to mitigate the risks associated with Bcc, a pathogen of increasing concern in healthcare settings.

## Figures and Tables

**Table 1:** Distribution of *Burkholderia* isolates by sample type and antimicrobial susceptibility.

	Material Type			Frequency (*n*)		Percent (%)
*Burkholderia cepacia*						
	Blood			59		76.6
	Endotracheal Aspirate			2		2.6
	Sputum			5		6.5
	BAL			1		1.3
	Urine			7		9.11
	Tissue/Wound/Fluid			3		3.9
	Total			77		100
*Burkholderia gladioli*	Endotracheal Aspirate			2		50
	BAL			1		25
	Urine			1		25
	Total			4		100
*Burkholderia cenocepacia*	Urine			1		100
Antimicrobial Susceptibility *n* (%)						
		CAZ	MEM	TXM-STX	TET	CHL
*Burkholderia cepacia*	S	3 (4.1)	66 (90.4)	2 (2.7)	0 (0)	0 (0)
	I	68 (93.2)	0	68 (93.2)	0 (0)	58 (95.1)
	R	2 (2.7)	7 (9.6)	3 (4.1)	68 (100)	3 (4.9)
	Total	73 (94.8)	73 (94.8)	73 (94.8)	68 (88.3)	61 (79.2)
	Missing	4 (5.2)	4 (5.2)	4 (5.2)	9 (11.7)	16 (20.8)
*Burkholderia cenocepacia*		R	R	I	R	missing
*Burkholderia gladioli*	S	0 (0)	3 (100)	0 (0)	0 (0)	0 (0)
	I	1	0	3 (100)	0	4 (100)
	R	2	0	0	4 (100)	0
	Total	3 (75)	3 (75)	3 (75)	4 (100)	4 (100)
	Missing	1 (25)	1 (25)	1 (25)	0 (0)	0 (0)

CAZ: Ceftazidime; MEM: Meropenem; TXM-STX: Trimethoprim/Sulphamethoxazole; TET: Tetracycline; CHL: Chloramphenicol; S: Sensitive; I: Intermediate; R: Resistance; BAL = Bronchoalveolar Lavage.

**Table 2:** Comparison of risk factors between *Burkholderia cepacia complex* cases and controls.

	No. (%) of Cases (*n* = 77)	No. (%) of Controls (*n* = 77)	*p*-Value	OR (%95CI)
Presence of risk factors				
Urinary catheter	54 (70.1)	49 (63.6)	0.494	1.34 (0.68–2.63)
Endotracheal intubation	24 (31.2)	23 (29.9)	>0.9	1.06 (0.54–2.11)
CVC	40 (51.9)	12 (15.6)	<0.001	5.86 (2.74–12.53)
PVC	72 (93.5)	76 (98.7)	0.209	0.19 (0.02–1.66)
PAC	25 (32.5)	12 (15.6)	0.023	2.60 (1.20–5.68)
Nasogastric tube	30 (39.0)	21 (27.3)	0.17	1.70 (0.86–3.36)
Chest tube	8 (10.4)	2 (2.6)	0.098	4.35 (0.89–21.19)
History of transfusion	28 (36.4)	13 (16.9)	0.01	2.81 (1.32–5.99)
Hemodialysis	4 (5.2)	2 (2.6)	0.681	2.06 (0.37–11.56)
Recent surgery	41 (53.2)	28 (36.4)	0.051	1.99 (1.05–3.78)
Surgical drain	15 (19.5)	10 (13)	0.382	1.62 (0.68–3.88)
Total parenteral nutrition	7 (9.1)	7 (9.1)	>0.9	1 (0.33–3.00)
Interventional radiology procedures	49 (63.6)	10 (13)	<0.001	11.73 (5.21–26.37)
Recent ICU admission	47 (61)	47 (61)	>0.9	1 (0.52–1.91)
Presence of pressure ulcers	3 (3.9)	1 (1.3)	0.62	3.08 (0.31–30.3)
Altered consciousness	30 (39)	19 (24.7)	0.083	1.95 (0.98–3.98)

Column percentages were calculated. CVC: Central Venous Catheter; PVC: Peripheral Venous Catheter; PAC: Peripheral Arterial Catheter; OR: Odds Ratio; CI: Confidence Interval; ICU: Intensive Care Unit. Results are reported as frequencies (*n*) and percentages (%). The categorical data associations were assessed using the Chi-square test or Fisher’s exact test, as appropriate. A *p*-value < 0.05 was considered as indicating statistical significance.

## Data Availability

The data sets generated during the current study are not publicly available due to the hospital’s data protection policy but are available from the corresponding author upon reasonable request. Additional Information: Correspondence and requests for materials should be addressed to H.N.K.P.

## References

[1] Jin Y., Zhou J., Zhou J., Hu M., Zhang Q., Kong N., Ren H., Liang L., Yue J. (2020). Genome-based classification of Burkholderia cepacia complex provides new insight into its taxonomic status. Biol. Direct.

[2] Govan J., Balendreau J., Vandamme P. (2000). Burkholderia cepacia—Friend and foe. ASM News.

[3] Kenna D.T.D., Lilley D., Coward A., Martin K., Perry C., Pike R., Hill R., Turton J.F. (2017). Prevalence of Burkholderia species, including members of Burkholderia cepacia complex, among UK cystic and non-cystic fibrosis patients. J. Med. Microbiol..

[4] Tunccan O.G., Dizbay M., Sezer B.E., Aktas F., Arman D. (2009). Nosocomial Burkholderia cepacia infections in a Turkish university hospital: A five-year surveillance. J. Infect. Dev. Ctries..

[5] Caraher E., Reynolds G., Murphy P., McClean S., Callaghan M. (2007). Comparison of antibiotic susceptibility of Burkholderia cepacia complex organisms when grown planktonically or as biofilm in vitro. Eur. J. Clin. Microbiol. Infect. Dis..

[6] Wiener-Well Y., Segonds C., Mazuz B., Kopuit P., Assous M.V. (2014). Successful outbreak investigation of Burkholderia cepacia complex bacteremia in intensive care patients. Am. J. Infect. Control.

[7] Becker S.L., Berger F.K., Feldner S.K., Karliova I., Haber M., Mellmann A., Schäfers H.-J., Gärtner B. (2018). Outbreak of Burkholderia cepacia complex infections associated with contaminated octenidine mouthwash solution, Germany, August to September 2018. Eurosurveillance.

[8] Tseng S.-P., Tsai W.-C., Liang C.-Y., Lin Y.-S., Huang J.-W., Chang C.-Y., Tyan Y.-C., Lu P.-L., Eberl L. (2014). The contribution of antibiotic resistance mechanisms in clinical burkholderia cepacia complex isolates: An emphasis on efflux pump activity. PLoS ONE.

[9] El Chakhtoura N.G., Saade E., Wilson B.M., Perez F., Papp-Wallace K.M., A Bonomo R. (2017). A 17-Year Nationwide Study of Burkholderia cepacia Complex Bloodstream Infections Among Patients in the United States Veterans Health Administration. Clin. Infect. Dis..

[10] Boszczowski I., Prado G.V.B.D., Dalben M.F., Telles R.C.P., Freire M.P., Guimaraes T., Oliveira M.S., Rosa J.F., Soares R.E., Llacer P.E.D. (2014). Polyclonal outbreak of bloodstream infections caused by Burkholderia cepacia complex in hematology and bone marrow transplant outpatient units. Rev. Inst. Med. Trop. Sao Paulo.

[11] Rhodes K.A., Schweizer H.P. (2016). Antibiotic resistance in Burkholderia species. Drug Resist. Updat..

[12] Al Zunitan M., Aldawood F., El-Saed A., Azzam M., Yassine K.A., Alshammari L., Alshamrani M. (2023). Two consecutive outbreaks caused by chlorhexidine mouthwash contaminated with Burkholderia contaminans in a two-hospital tertiary care system. J. Hosp. Infect..

[13] Bharara T., Chakravarti A., Sharma M., Agarwal P. (2020). Investigation of Burkholderia cepacia complex bacteremia outbreak in a neonatal intensive care unit: A case series. J. Med. Case Rep..

[14] Marquez L., Jones K.N., Whaley E.M., Koy T.H., Revell P.A., Taylor R.S., Bernhardt M.B., Wagner J.L., Dunn J.J., LiPuma J.J. (2017). An Outbreak of *Burkholderia cepacian* Complex Infections Associated with Contaminated Liquid Docusate. Infect. Control Hosp. Epidemiol..

[15] Lee Y.-M., Park K.-H., Moon C., Kim D.Y., Lee M.S., Kim T., Choo E.J., Chong Y.P., Kim S.-H., Kim Y.S. (2020). Management and outcomes of *Burkholderia cepacia* complex bacteremia in patients without cystic fibrosis: A retrospective observational study. Eur. J. Clin. Microbiol. Infect. Dis..

[16] Bressler A.M., Kaye K.S., LiPuma J.J., Alexander B.D., Moore C.M., Reller L.B., Woods C.W. (2007). Risk factors for *Burkholderia cepacian* complex bacteremia among intensive care unit patients without cystic fibrosis: A case-control study. Infect. Control Hosp. Epidemiol..

[17] Shi H., Chen X., Chen L., Zhu B., Yan W., Ma X. (2023). Burkholderia cepacia infection in children without cystic fibrosis: A clinical analysis of 50 cases. Front. Pediatr..

[18] Lee J.K. (2008). Two outbreaks of *Burkholderia cepacia* nosocomial infection in a neonatal intensive care unit. J. Paediatr. Child Health.

[19] Dolan S.A., Dowell E., LiPuma J.J., Valdez S., Chan K., James J.F. (2011). An outbreak of Burkholderia cepacia complex associated with intrinsically contaminated nasal spray. Infect. Control Hosp. Epidemiol..

[20] Glowicz J., Crist M., Gould C., Moulton-Meissner H., Noble-Wang J., de Man T.J., Perry K.A., Miller Z., Yang W.C., Langille S. (2018). A multistate investigation of health care–associated Burkholderia cepacia complex infections related to liquid docusate sodium contamination, January-October 2016. Am. J. Infect. Control.

[21] Bovolenta E., Zanon M.P., Mondino S., Manfrin V., Rassu M., Diquigiovanni A., Zenere A., Bertoncello C., Cazzaro R., Direction V.R.M.-H.M. (2023). Management of an Epidemic Outbreak of Burkholderia Cepacia in A Hospital in The North of Italy. J. Food Nutr..

[22] Paraskevopoulos D.K.d.S., Camargo C.H., Kodato P.K., Yamada A.Y., Almodovar A.A.B., Hilinski E.G., de Paula A.I., Irineu E.F., Barrio S.R., Fonseca C.L. (2025). A Burkholderia contaminans outbreak in an intensive care unit associated with contaminated bath solution: Control and microbiological findings. Am. J. Infect. Control.

[23] Ormsby J., Wagner T., Gupta R., Millson T., Phillips B. (2025). Safe injection, infusion, medication vial, and point-of-care testing practices in health care (2025). Am. J. Infect. Control.

[24] World Health Organization (2016). Guidelines on Core Components of Infection Prevention and Control Programmes at the National and Acute Health Care Facility Level.

[25] Centers for Disease Control and Prevention (CDC) (2024). Core Infection Prevention and Control Practices for Safe Healthcare Delivery in All Settings.

[26] Ibrahim T., Abdallah T.A., Abdallah A., Qazi R., Alimam A., Mohammad H., Eltayeb F., Daghfal J., Ali M., Hadi H.A. (2024). Epidemiology, microbiological, clinical characteristics, and outcome of *Burkholderia cepacia* complex infections in non-cystic fibrosis adult patients from Qatar. IJID Reg..

[27] Nazik S., Topal B., Şahin A.R., Ateş S. (2019). Nosocomial *Burkholderia cepacia* infection in a tertiary hospital; Five-year surveillance: A retrospective cross-sectional study. J. Surg. Med..

[28] Cheesbrough M. (2005). District Laboratory Practice in Tropical Countries. Part 2.

[29] Ransom E.M., Alipour Z., Wallace M.A., Burnham C.-A.D., Simner P.J. (2021). Evaluation of Optimal Blood Culture Incubation Time To Maximize Clinically Relevant Results from a Contemporary Blood Culture Instrument and Media System. J. Clin. Microbiol..

[30] Baselski V., Klutts J.S., Gilligan P.H. (2013). Quantitative Cultures of Bronchoscopically Obtained Specimens Should Be Performed for Optimal Management of Ventilator-Associated Pneumonia. J. Clin. Microbiol..

[31] Hasanin A., Mukhtar A., El-Adawy A., Elazizi H., Lotfy A., Nassar H., Ghaith D. (2016). Ventilator associated pneumonia caused by extensive-drug resistant *Acinetobacter* species: Colistin is the remaining choice. Egypt. J. Anaesth..

[32] The European Committee on Antimicrobial Susceptibility Testing (EUCAST) (2025). Breakpoint Tables for Interpretation of MICs and Zone Diameters. Version 15.0. https://www.eucast.org/fileadmin/src/media/PDFs/EUCAST_files/Breakpoint_tables/v_15.0_Breakpoint_Tables.pdf.

